# Content-Based Multi-Channel Network Coding Algorithm in the Millimeter-Wave Sensor Network

**DOI:** 10.3390/s16071023

**Published:** 2016-07-01

**Authors:** Kai Lin, Di Wang, Long Hu

**Affiliations:** 1 School of Computer Science and Technology, Dalian University of Technology, Dalian 116024, China; dlut_wd@mail.dlut.edu.cn; 2 School of Computer Science and Technology, Huazhong University of Science and Technology, Wuhan 430074, China; longhu.cs@gmail.com

**Keywords:** millimeter-wave sensor network, network coding, multi-channel assignment, data fusion, data content

## Abstract

With the development of wireless technology, the widespread use of 5G is already an irreversible trend, and millimeter-wave sensor networks are becoming more and more common. However, due to the high degree of complexity and bandwidth bottlenecks, the millimeter-wave sensor network still faces numerous problems. In this paper, we propose a novel content-based multi-channel network coding algorithm, which uses the functions of data fusion, multi-channel and network coding to improve the data transmission; the algorithm is referred to as content-based multi-channel network coding (CMNC). The CMNC algorithm provides a fusion-driven model based on the Dempster-Shafer (D-S) evidence theory to classify the sensor nodes into different classes according to the data content. By using the result of the classification, the CMNC algorithm also provides the channel assignment strategy and uses network coding to further improve the quality of data transmission in the millimeter-wave sensor network. Extensive simulations are carried out and compared to other methods. Our simulation results show that the proposed CMNC algorithm can effectively improve the quality of data transmission and has better performance than the compared methods.

## 1. Introduction

With the development of wireless communication technology in recent years, more and more applications have the characteristics of being wireless and mobile [1]. Increasing devices and the amount of data growth bring the plight of the shortage of bands to the wireless sensor networks. The emergence of 5G technology provides a good solution for us, which has the advantages of high-speed transmission, low latency and anti-interference [2,3]. Specifically, one of the most promising potential 5G technologies under consideration is the millimeter-wave, which allocates more bandwidth to provide faster, higher-quality data transmission. The previous articles [4,5,6] demonstrate the huge advantages of utilizing millimeter-wave in various applications. Considering the increasing number of devices in wireless sensor networks, these advantages bring more stringent design requirements on developing new sensor nodes equipped with a communication model based on the millimeter-wave. Therefore, the millimeter-wave is considered to be a valuable support technology of wireless sensor networks, and the emerging millimeter-wave sensor network has great potential in future applications [7,8].

However, there are significant differences between traditional wireless media and millimeter-wave at the physical (PHY), media access control (MAC) and network layers, which means the requirement of new designs. Propagation and path loss, antenna directivity, sensitivity to blockage and the dynamic topology of millimeter-wave require new ideas and protocols to ensure the performance of communication. Since the millimeter-wave has a high carrier frequency, the sensor nodes in the millimeter-wave sensor network have a smaller communication range than that of the traditional sensor network, which encourages the tasks to be migrated seamlessly across different regions by using the distribution mechanism. Considering the high spatial multiplexing and spectral efficiency, various sensor nodes often transmit data simultaneously, which need to be coordinated in the millimeter-wave network on multiple interfering links [5]. Therefore, how to achieve high efficiency and stable data transmission in the millimeter-wave sensor network is still a big challenge.

Many researchers have proposed various methods to improve the performance of data transmission in the wireless network. Mekikis et al. [9] studied the information change of the wireless energy harvesting-enabled sensor nodes in the dense network and provided the theoretical expressions for the probability of successful communication in the different communication scenarios such as direct and cooperative. Murali et al. [10] proposed a method of using network coding (NC) to solve unreliable individual communications in millimeter-wave systems, which uses a random linear network coding (RLNC) to compare with traditional routing methods. In order to provide the energy efficient solutions to the terminal in the network, two game theoretic strategies [11] were introduced to solve the conflicts caused by the sensor nodes. The two strategies balance the relationship between completing the data dissemination and conserving energy. Otherwise, NC was utilized to take away the need of control packet exchange. Shafieinejad et al. [12] designed a network coding and multi-channel/interface cooperative model under a wireless network environment, which includes the integration of network coding, routing and channel assignment. A Star-NC was also developed to take full advantage of aggregate throughput. Sun et al. [13] proposed a Multiple-Input Multiple-Output Dempster-Shafer Network Coding (MIMO-DS NC) program using Dempster-Shafer (D-S) evidence theory to detect signals for each source node before mapping the source signals into a network-coded signal. An MIMO-DS physical-layer network coding (PNC) program was also further proposed from the direction of the vector space, which does not need estimating the source node signal before obtaining the network-coded signal in combination with PNC mapping and D-S evidence theory. However, the content of the data is ignored in the above designs, which greatly affects the performance of data transmission in the millimeter-wave network.

In this paper, we focus on how to improve the performance of data transmission in the millimeter-wave sensor network. Firstly, a fusion-driven model is proposed based on the D-S evidefig2nce theory method to integrate different sensory data by exploring the relationship between them, which aims to obtain accurate data results and remove the redundant data during the data collection. D-S is a mathematical theory based on the plausible reasoning and belief functions. It was first introduced by Dempster [14] in 1967 and developed in 1970 by Shafer [15]. It has good application in many areas such as remote sensing classification [16], medical imaging [17] and object detection [18]. Considering the function of solving the uncertainty problem with uncertain and imprecise information, we utilize the D-S evidence theory as a framework to classify the sensor nodes based on the correlation between the sensory data. Secondly, based on the fusion-driven model, we proposed a novel content-based multi-channel network coding (CMNC) algorithm. In the CMNC algorithm, the sensor nodes with related data are assigned to the same channel, which effectively improves the quality of data transmission by realizing the functions of both data fusion and network coding during data collection. To the best of our knowledge, it is the first work to study both the data fusion problem and the network coding problem for multi-channel data transmission in the millimeter-wave sensor network. This paper offers the following contributions addressing the issues mentioned above:

- We design a fusion-driven model based on the D-S evidence theory to classify the sensor nodes according to the content of their data. By using this model, the sensor nodes with related data are classified for further processing to obtain more accurate data results and remove the redundant data during the data collection.
- We propose the CMNC algorithm, which combines the use of data fusion and network coding for multi-channel data transmission in the millimeter-wave sensor network. The obtained content relevance of the sensory data is adopted for channel assignment to fully utilize the functions of data fusion and network coding.
- We perform extensive simulations to evaluate the proposed CMNC algorithm by comparing to other algorithms under several performance criteria. Simulation results demonstrate that the CMNC algorithm achieves high performance for data transmission in the millimeter-wave sensor network.

The remainder of this paper is organized as follows. Section 2 presents some related work. Section 3 introduces the system model and problem statement. Section 4 describes the fusion-driven model based on D-S evidence theory. The proposed CMNC algorithm is given in Section 5. In Section 6, we evaluate the performance of the CMNC algorithm based on simulations. The paper is concluded in Section 7.

## 2. Related Work

Currently, the millimeter-wave sensor network has been considered as an important part of 5G technology and can provide essential support for data collection. Developing new methods for the millimeter-wave sensor network has attracted more attention from diverse perspectives. The related works according to our considerations can be divided into three aspects: data fusion, network coding, and wireless multi-channel assignment.

Data fusion technology is valuable for obtaining more precise results while eliminating redundant data. D-S evidence theory [14,15,19] was proposed and proven to have high performance in many areas. For example, in the cooperative communication field, Qin et al. [20] explained how to use the D-S evidence theory in a cellular network, relying on the advantages of multi-channel sources using the D-S evidence theory to achieve a more reliable statistical decision. Kessentini et al. [21] used the D-S theory to improve the accuracy and reliability of a handwriting recognition system. Dong et al. [22] then introduced the D-S theory in the direction of target recognition, the paper proposes a monogenic signal of the multidimensional analytic signal utilizing the D-S theory to solve the problem, which cannot be used directly.

NC was used in [23] for the first time, which is a kind of combination by passing packets to improve the throughput of multicast applications. Recently, network coding has had an excellent development in many areas. For instance, in order to solve the problem of different packet length for the network coding, Torres Compta et al. [24] designed a series of mechanisms to manage heterogeneous data packet length and used the real packet length distribution to analyze the mechanism of the induction of overhead. Leu et al. [25] proposed a sharing system in P2P networks based on network coding with considering the compilation, decoding delay and message overhead. Jiang et al. [26] established a multicast routing algorithm based on network coding multi-hop wireless networks, the algorithm can increase energy efficiency and throughput. Antonopoulos et al. introduced a novel NC-aided MAC protocol [27] in the automatic repeat request-based wireless network. A cross-layer analytical model was further designed to solve such a problem in correlated shadowing conditions [28].

Multi-channel assignment focuses on solving the problem of channel contention in order to ensure the performance of data transmission. Lin et al. [29] had proposed a green video transmission algorithm, which combines the video clustering and channel assignment to assist in video transmission. Wu et al. [30] investigated the multichannel feasibility in the sensor network and designed a multi-channel scheme, which assigns the channels based on the tree structure while realizing parallel data transmission. Phung et al. [31] aimed at decreasing the influences of the interference caused by the channel contention, the limited radio frequency bandwidth and the limited battery power of sensor nodes. They explored the wireless multi-channel operational ability to provide higher network throughput and proposed a non-coordinated transmissions scheduling algorithm to eliminate the conflict, the idle listening and excessive hearing.

## 3. System Model and Problem Statement

### 3.1. System Model

In this paper, we assume a wireless sensor network supported by the millimeter-wave technology, which provides plenty of reliable services to users. In our research, the sensor nodes are resource-constrained and under the millimeter-wave sensor network environment. The ordinary node and the sink node constitute the entire sensor network. These sensor nodes have very strong adaptability and ductility while accessing the network effortlessly and communicating with their neighbors. One sink node is deployed as the destination of data collection in the millimeter-wave sensor network, which has no limitation of computation and communication. The biggest advantage of the sensor network, the complexity of data processing, access processing and data transmission will be completed by the sink node. The network is responsible for transmitting a large amount of information to meet the requirements of applications. Each sensor node can exchange data with other sensor nodes located in its communication range. At the same time, the sensor nodes can execute data process functions for both their own generated data and received data from other sensor nodes.

Considering that the multi-channel technology is adopted in the millimeter-wave sensor network, we assume that these sensor nodes can communicate properly with their neighbors that use the same channel for data transmission. Data fusion is a work of great significance, which benefits the whole network during the data transmission process between different sensor nodes. We explore the data relativity between the sensor nodes for further processing to reduce the overload of the network while obtaining precise results. Meanwhile, network coding is also considered in our research, which is able to encode data that are transported to a common sensor node. According to the network topology, the nodes belong to different transmission paths and can encode multiple data to increase transmission throughput.

Without loss of generality, we assume that the sensor nodes are diverse, which means they differ in storage capacity, transmission speed, communication range, computing power, etc. All communication links are symmetrical. According to the distance to the receiver, the sensor nodes can adjust the transmission power to save energy consumption [32,33]. Each sensor node has the ability of data fusion, and network coding then exchanges the result with its neighbors using the same channel. The only difference between our system model and traditional sensor networks is that the communication model uses the millimeter-wave instead of the radio frequency. This change decreases the sensor nodes’ neighbors because the communication range of millimeter-wave is shorter than that of radio frequency. The algorithm designed in this article need to consider the effect caused by this difference, but it can also be carried out in the majority of the traditional sensors.

To further coordinate sensor nodes’ communication in the millimeter-wave sensor network, these sensor nodes are considered to be classified into different classes according to the content of their generated data. The set of classes is denoted as $\omega =\left\{\omega_{1},\omega_{2},\ldots ,\omega_{M}\right\}$, where *M* is the number of classes. Before data transmission, each sensor node selects the appropriate channel based on the class information, while the relay sensor nodes, adopting the same channel, encode and fuse the data and transmit the result to the destination sensor node. For example, the data $x_{1}$ are generated by a sensor node belonging to the class $\omega_{1}$, and the data $x_{2}$ are generated by another sensor node belonging to the class $\omega_{2}$. These two sensor nodes are assigned different channels $C_{1}$ and $C_{2}$. In channel $C_{1}$, if there exists data $x_{1}^{\prime }$ that occupies the channel $C_{1}$ to transmit, a collision may occur in the relay sensor node during the transmission. With the increasing number of transmitted data in the network, the shortage becomes obvious as heavy overload shares the same channel simultaneously.

### 3.2. Problem Statement

For the disordered data transmission in the millimeter-wave sensor network, the existing redundant data lead to a waste of resources. Improving resource utilization by data processing is the focus of our concern. The sensor nodes are able to generate and receive data with varying content, such as temperature, humidity, sound, etc. These different data are often transmitted at the same time, which causes heavy congestion and jams in the network and affects the performance of the end-to-end transmission. Processing these data reasonably during the data collection is very important for the millimeter-wave sensor network. Considering that the millimeter-wave has a short distance of communication, the end-to-end data transmission needs to be delivered through multiple routes in a multi-hop way. In the process of routing transfer, network coding is introduced to further improve the performance of data transmission.

**Figure 1:**
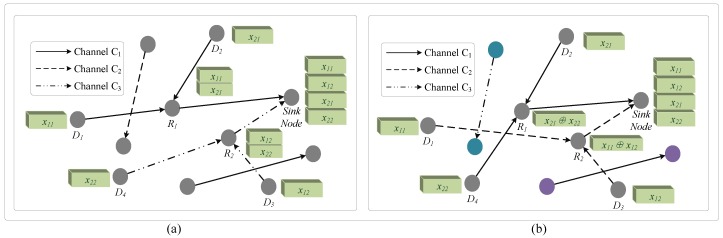
Different methods to transmit data with millimeter-wave technology. (**a**) Traditional transmission method with random channel assignment; (**b**) the transmission using the content-based multi-channel network coding (CMNC) algorithm.

Each sensor node can send exchange data to other sensor nodes adopting the same channel through multiple routes. As shown in Figure 1, each block represents a kind of data in the network, and each sensor node has a data block. Figure 1a shows a traditional transmission method with random channel assignment; the relay nodes $R_{1},\;R_{2}$ receive the data from $D_{1},\;D_{2}$ and $D_{3},\;D_{4}$, respectively, then forward the data to the sink node. $D_{1},\;D_{2}$ transmit the data $x_{11},\;x_{21}$ to the sink node sequentially through a selected relay node $R_{1}$, while $D_{3}$ and $D_{4}$ send data $x_{12},\;x_{22}$ through the relay node $R_{2}$. We assume that data $x_{11}$ are relevant to data $x_{12}$ and data $x_{21}$ are relevant to data $x_{22}$. However, these relevant data cannot be processed by both data fusion and network coding at the relay node to eliminate unnecessary data transmission because they are transmitted differently. To solve this problem, we consider dividing the sensor nodes into different classes according to their data content and assign the same channel for the sensor nodes in the same class. Compared to the traditional approach, the advantage of this method is to decrease the redundant data, improve the channel utilization efficiency and reduce channel congestion during the data transmission. The relay sensor node can process data fusion and network coding on different data during data transmission.

In this paper, our objective is designing an optimal strategy to improve the efficiency of data transmission in the millimeter-wave sensor network. The first challenge is how to classify the sensor nodes according to their data content. By introducing D-S evidence theory, we classify the sensor nodes based on the data content by exploring the relativity between their data. The performance of data transmission can be enhanced by the process of data fusion. On the other hand, network coding is also adopted to further improve the throughput of the millimeter-wave sensor network. We use RLNC to encode the data transmitted to the same terminal sensor node and decrease the number of transmissions. Multiple data are encoded by the relay sensor node to one joint transmission for reducing the occupation of the spectrum. Specifically, the channel assignment method is designed to ensure that the functions of data fusion and network coding can be realized during the data transmission. The sensor nodes with related data are allocated the same channel for data transmission. Optimal channel assignment is capable of reducing transmission congestion and improves the channel utilization. Therefore, our objective is realized on the above factors, including data fusion, network coding and channel assignment.

## 4. The Fusion-Driven Model Based on D-S Evidence Theory

This section presents a fusion-driven model base on D-S evidence theory, which classifies the sensor nodes into different classes depending on their data content. The results of sensor node classification can be used to improve the quality of data transmission in the millimeter-wave sensor network.

### 4.1. Basic Mathematical Terminology of D-S Evidence Theory

The theory of evidence is also called Dempster–Shafer theory or evidence reasoning. The D-S evidence theory aims to make a decision based on the evidence for solving the uncertainty problem with uncertain and imprecise information. The evidence here means the result of the issues from the observation and research, which can provide a required belief value for making the decision in the D-S evidence theory. In this paper, we want to classify the sensor nodes by exploring the correlation between the sensory data from different sensor nodes, which is a typical uncertainty problem because it has the characteristics of imprecision and uncertainty. The D-S evidence theory can solve such a problem and effectively classify the sensor nodes according to the judgment of data correlation.

In practical applications, the evidence is carried out in so many different ways, such as decision-makers’ experience, knowledge and observation. Although a function can be built to generate the evidence from only one decision-maker who is trusted and influential, the limitation of individual ability reduces the validity and usability of the evidence. Therefore, the evidence combination rule is introduced as a powerful tool in the D-S evidence theory to integrate multiple opinions from decision-makers in order to get more accurate results. In this paper, the sensor nodes act as the decision-makers.

Let us define Φ, a frame of discernment, which is a finite set of mutually exclusive propositions and hypotheses about some problem domains. Only by establishing a reasonable frame of discernment, we can carry out the research of the proposition converted into the study of function and probability assignment. Φ is the set of all considerations on the case and needs to cover all possible problems. In order to classify the sensor nodes according to the decision made by the D-S evidence theory, the frame of discernment represents the class set in the millimeter-wave sensor network, while each sensor node belongs to the existing class at any time. The *power set* $2^{\Phi }$ is the set of all possible subsets Φ, including the empty set ∅. For example, if:

(1) Φ={a,b,c}

this means three classes a,b,c in the network, then:

(2) $$2^{\Phi }=\left\{\emptyset ,\left\{a\right\},\left\{b\right\},\left\{c\right\},\left\{a,b\right\},\left\{b,c\right\},\left\{a,c\right\},\Phi \right\}$$

That means a sensor node can belong to more than one class. The mathematical definition of ∅ represents that a sensor node does not belong to any class. To meet the requirement of algorithm design and network operation, we remove the situation of the ∅ in this work, and each sensor node can only belong to one class at a time.

Each sensor node establishes its observation by identifying its belief on the frame of discernment Φ. The belief represents the judgment to which class the sensor node belongs. The D-S evidence theory defines a belief value *m*, which ranges from zero to one and can be expressed as:

(3) $$m:2^{\Phi }\to \left[0,1\right]$$

This assignment function is called the *basic probability assignment* (BPA or sometimes the *mass function*). A *basic probability assignment* is a function *m* from $2^{\Phi }$, the power set of Φ, to [0,1]. *A* represents any subset built on a frame of discernment Φ and associated with a corresponding proposition, referred to as A⊆Φ. Verifying:

(4) m(∅)=0

(5) $$\sum_{A\subseteq \Phi }m\left(A\right)=1$$

The quantity m(A), called a *basic probability number* or *mass value*, is the measure of the probability that one is willing to commit exactly to *A* without any subsets, which offers a certain piece of evidence. This means the probability of a node belonging to the class. The condition of complete ignorance is characterized by m(Φ)=1.

According to the BPA from the power set, the upper and lower boundary of the probability interval can be determined and named the *degree of belief (Bel)* and *plausibility (Pl)*. In order to obtain a total belief in *A*, one must add to m(A) the quantities m(B) for all subsets *B* of *A*. There is a function assigned to each subset *A* of Φ, and the total number of all basic probability numbers for subsets of *A* is the *belief function*:

(6) $$Bel\left(A\right)=\sum_{B\subseteq A}m\left(B\right)$$

Bel(A) is also called the *credibility* of *A* and is considered as a total belief committed to *A*. Considering that *A* represents the subsets of Φ, m(A)>0 is the *focal elements* of the belief function. A set containing all focal elements is called the *core* of the belief function.

Compared to probability theory, it is easy to verify that the belief in some hypothesis *A* and its negation $\bar{A}$ does not necessarily sum to one. Bel(A) does not reveal the degree of belief in $\bar{A}$, i.e., the degree of doubt of *A*, which is described by $Bel(\bar{A})$. The quantity $Pl\left(A\right)=1-Bel\left(\bar{A}\right)$ represents the *plausibility* of *A* and defines the degree to which one fails to doubt in *A*, i.e., the *A* is considered as plausible. It is shown that:

(7) $$Pl\left(A\right)=\sum_{B⋂A\neq \emptyset }m\left(B\right)$$

If any one of these three functions *m*, Bel and Pl is sufficient to cover the other two, it follows from the definition of Pl(A) as $1-Bel(\bar{A})$, and:

(8) $$m\left(A\right)=\sum_{B\subseteq A}{\left(-1\right)}^{\left|A\backslash B\right|}Bel\left(B\right)$$

Interval [Bel(A),Pl(A)] represents the uncertain interval, which means neither supporting nor rejecting the proposition *A*. Bel(A) and Pl(A) are also called lower and upper probabilities, where:

(9) Bel(A)≤m(A)≤Pl(A)

**Figure 2:**
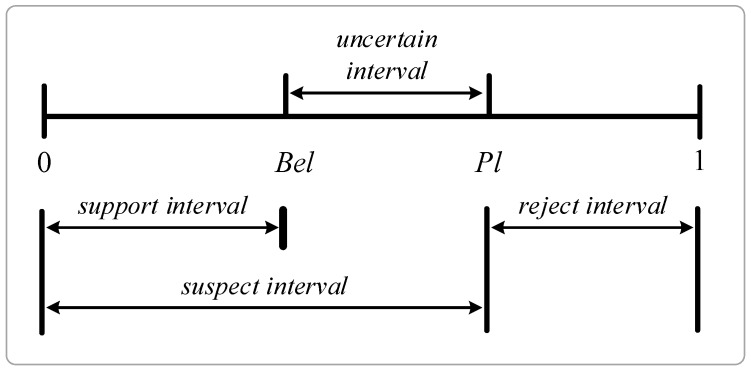
Uncertainty representation of the information.

The larger Bel(A) indicates a higher degree of belief, which means the sensor node is more likely to belong to the class. The relationships between the Bel(A) value, the Pl(A) value and the uncertain interval are shown graphically in Figure 2 [14,15].

For the operations of the use of D-S evidence theory, more BPA are given under the same frame of discernment. If the evidence is not entirely in conflict, thus it goes through a reasonable method to combine or integrate the multiple BPA. The D-S evidence theory utilizes the orthogonal to combine multiple BPA for building a new function, named *Dempster’s rule of combination*. The new function can be thought of as some evidence under the joint action producing a new belief for BPA, and it combines multiple functions under the same frame of discernment. In each classification period, a sensor node to be classified can obtain the BPA from classified sensor nodes and make a better decision for classification by using an updated BPA generated by comprehensive information.

Firstly, we introduce the combination rule of D-S evidence theory from two pieces of evidence. Assume that there are two pieces of evidence $E_{1}$ and $E_{2}$ on the frame of discernment; $m_{1}$ and $m_{2}$ are the BPA of the two pieces of evidence. $A_{1}$ and $A_{2}$ are the focus elements of $E_{1}$ and $E_{2}$, respectively. Belief functions $Bel_{1}$ and $Bel_{2}$ under the same frame of discernment are composed of two independent sources of information. D-S evidence theory provides a convenient method to integrate the evidence. The premise that $Bel_{1}$ and $Bel_{2}$ are able to combine into a single one is based on their cores having to intersect. This condition can be expressed as follows:

(10) $$\sum_{A_{1}⋂A_{2}=\emptyset }m_{1}\left(A_{1}\right)m_{2}\left(A_{2}\right)<1$$

The combined BPA of $m_{1}$ and $m_{2}$ (the joint mass) is:

(11) m(∅)=0

(12) $$m\left(A\right)=\frac{\sum_{A_{1}⋂A_{2}=A}m_{1}\left(A_{1}\right)m_{2}\left(A_{2}\right)}{1-K}$$

where *K* is given by:

(13) $$K=\sum_{A_{1}⋂A_{2}=\emptyset }m_{1}\left(A_{1}\right)m_{2}\left(A_{2}\right)$$

It reflects the degree of conflict between the various evidence; the coefficient 1/(1−K) is known as the *normalization factor*.

Belief function Bel is given by *m*, called the orthogonal sum of $Bel_{1}$ and $Bel_{2}$, while expressed as $Bel_{1}⨁Bel_{2}$. For convenience, *m* is represented as $m_{1}⨁m_{2}$. The *Bel’s* core is the intersection of $Bel_{1}$ and $Bel_{2}$. If the $m_{1}$ and $m_{2}$ cores are disjointed, $m_{1}$ and $m_{2}$ cannot be combined, and their corresponding evidence supports a different proposition, which means the two pieces of evidence are entirely in conflict. The two pieces of evidence cannot be combined because there is no intersection. For example, if one piece of evidence supports the proposition while another piece of evidence supports the non-proposition, such two pieces of evidence cannot be combined.

As an example of Dempster’s rule of combination, there are mass values in the set: $m_{1}$ are $m_{1}\left(a_{1}\right)=0.3$, and $m_{1}\left(a_{2}\right)=0.7$. Additionally, the other mass values in the set $m_{2}$ are $m_{2}\left(a_{1}\right)=0.4$ and $m_{2}\left(a_{2}\right)=0.6$, where $a_{1}$ and $a_{2}$ represent the that the hypothesis is normal and abnormal. We first calculated the normalization factor *K* and summed the results:

(14) $$K=\left(m_{1}\left(a_{1}\right)\times m_{2}\left(a_{2}\right)\right)+\left(m_{1}\left(a_{2}\right)\times m_{2}\left(a_{1}\right)\right)=\left(0.3\times 0.6\right)+\left(0.7\times 0.4\right)=0.46$$

Then, the results of the joint masses are:

(15) $$m\left(a_{1}\right)=\frac{1}{1-0.46}\times m_{1}\left(a_{1}\right)\times m_{2}\left(a_{1}\right)=\frac{2}{9}$$

and:

(16) $$m\left(a_{2}\right)=\frac{1}{1-0.46}\times m_{1}\left(a_{2}\right)\times m_{2}\left(a_{2}\right)=\frac{7}{9}$$

### 4.2. Sensor Node Classification Based on D-S Evidence Theory

In this section, we present the fusion-driven model based on D-S evidence theory. Considering that the data content is the key factor of data fusion, the sensor nodes are divided into different classes according to different contents of the stored data for further processing.

We define $\vartheta =\{D=\left(D_{i}^{1},\ldots ,D_{i}^{P}\right)|i=1,\ldots ,Q\}$ as a set of Q P-dimensional training samples and $\omega =\left\{\omega_{1},\omega_{2},\ldots ,\omega_{M}\right\}$ as a collection of the classes. Each sensor node has a class symbol $L^{i}\in \left\{1,\ldots M\right\}$, which indicates each sensor node belonging to different classes *ω*. (ϑ,L) is formed on a training set to classify the new model, where *L* is a collection of labels.

Our approach is to identify all of the classes as a frame of discernment, which is set in the classification problem to recognize all of the possible classification results. The *ω* is called frame of discernment Φ to explain the issues by D-S theory. $D_{s}$ is a sample to be classified by the information of the training set. $\xi^{s}$ is defined as a sensor node $D_{s}$ that may belong to the set of the same class in *ϑ* depending on the classification of its data contents $\delta =\{\delta_{D_{1}}^{x_{1}},\ldots ,\delta_{D_{n}}^{x_{r}}\}$, and the number of sensor nodes in $\xi^{s}$ is *η*, where *n* represents the number of sensor nodes and *r* is the number of data blocks in the sensor node. The amount of data *r* in the sensor node obeys the Poisson distribution. The function of the data content *δ* is given by:

(17) $$\mathrm{Possion}\left(\delta^{x_{r}}=k\right)=\frac{\lambda^{k}exp\left(-\lambda \right)}{k!}$$

where λ>0.

For any $D_{i}\in \xi^{s}$, $L^{i}=q$ is considered as an increase of the possible belief that $D_{s}$ also belongs to $\omega_{q}$. However, the evidence does not provide one hundred percent of the confirmation. In the D-S evidence theory, this can be expressed as only a portion of our belief belonging to $\omega_{q}$. Therefore, $L^{i}=q$ cannot point out another hypothesis, which means the rest of our belief except *ω* cannot be assigned to others. This evidence behaves as the BPA:

(18) $$m^{s,i}\left(\left\{\omega_{q}\right\}\right)=\theta \phi_{q}\left(\delta_{i}\right)$$

(19) $$m^{s,i}\left(\Phi \right)=1-m^{s,i}\left(\left\{\omega_{q}\right\}\right)$$

(20) $$m^{s,i}\left(A\right)=0,\forall A\in 2^{\Phi }\backslash \left\{\Phi ,\left\{\omega_{q}\right\}\right\}$$

*φ* is an increasing function, value of which in [0,1] and *θ* is the value of the interval parameters. If $D_{i}$ and $D_{s}$ have large difference in data contents, class $D_{i}$ is considered to provide little information about the $D_{s}$ class. On the contrary, if the data contents between $D_{i}$ and $D_{s}$ have a lot of relevancy, they tend to belong to the same class between $D_{i}$ and $D_{s}$. As a consequence, *φ* is obtained by the data content *δ* with $\gamma_{q}$ and β={1,2,…}.

(21) $$\phi_{q}\left(\delta \right)=exp\left(\gamma_{q}\delta_{i}^{\beta }\right)$$

*β* usually chooses a small value (one or two). The index *q* shows that the influence of $\delta_{i}$ is affected by the class of $D_{s}$.

For the *η* sensor nodes having relevant content included by $D_{s}$, a BPA is defined by its class label and the relatedness of data content with $D_{s}$. In order to determine to which class a sensor node belongs, we use D-S evidence theory to combine these BPAs. It is always possible that all of the belief functions use *ω* as the focal element.

For example, we first consider that $D_{i}$ and $D_{j}$ of $\xi^{s}$ belong to the same class $\omega_{q}$. The BPA $m^{s,(i,j)}$ is a combination of two BPA functions $m^{s,i}$ and $m^{s,j}$, respectively.

(22) $$m^{s,(i,j)}\left(\left\{\omega_{q}\right\}\right)=1-\left(1-m^{s,i}\left(\left\{\omega_{q}\right\}\right)\right)\left(1-m^{s,j}\left(\left\{\omega_{q}\right\}\right)\right)$$

(23) $$m^{s,(i,j)}\left(\Phi \right)=\left(1-m^{s,i}\left(\left\{\omega_{q}\right\}\right)\right)\left(1-m^{s,j}\left(\left\{\omega_{q}\right\}\right)\right)$$

Other evidence’s belief assignment can be created based on the combination that has been generated. Thus, all of the beliefs are reallocated after the combination of evidence, and the global combined belief assignment is finally obtained. By combining each class’s BPA $m_{q}^{s}$, a global BPA $m^{s}={⊕}_{q=1}^{M}m_{q}^{s}$ is defined as:

(24) $$m^{s}\left(\left\{\omega_{q}\right\}\right)=\frac{m_{q}^{s}\left(\left\{\omega_{q}\right\}\right)\prod_{z\neq q}m_{z}^{s}\left(\Phi \right)}{\sum_{q=1}^{m}m_{q}^{s}\left(\left\{\omega_{q}\right\}\right)\prod_{z\neq q}m_{z}^{s}\left(\omega \right)+\prod_{q=1}^{M}m_{q}^{s}\left(\Phi \right)}$$

(25) $$m^{s}\left(\Phi \right)=\frac{\prod_{q=1}^{M}m_{q}^{s}\left(\Phi \right)}{\sum_{q=1}^{m}m_{q}^{s}\left(\left\{\omega_{q}\right\}\right)\prod_{z\neq q}m_{z}^{s}\left(\Phi \right)+\prod_{q=1}^{M}m_{q}^{s}\left(\Phi \right)}$$

where the normalization factor *K* is:

(26) $$\begin{array}{c} K=\sum_{q=1}^{m}m_{q}^{s}\left(\left\{\omega_{q}\right\}\right)\prod_{z\neq q}m_{z}^{s}\left(\Phi \right)+\prod_{q=1}^{M}m_{q}^{s}\left(\Phi \right) \end{array}$$

(27) $$\begin{array}{c} =\sum_{q=1}^{m}\prod_{z\neq q}m_{z}^{s}\left(\Phi \right)+\left(1-M\right)\prod_{q=1}^{M}m_{q}^{s}\left(\Phi \right) \end{array}$$

The credibility $Bel^{s}\left(\left\{\omega_{q}\right\}\right)$ and plausibility $Pl^{s}\left(\left\{\omega_{q}\right\}\right)$ are calculated by a given class $\omega_{q}$:

(28) $$Bel^{s}\left(\left\{\omega_{q}\right\}\right)=m^{s}\left(\left\{\omega_{q}\right\}\right)$$

(29) $$Pl^{s}\left(\left\{\omega_{q}\right\}\right)=m^{s}\left(\left\{\omega_{q}\right\}\right)+m^{s}\left(\Phi \right)$$

In the case of {0,1} costs without refusal decisions, Bayes’ rule always selects a class with a maximum posterior probability. We select a maximum credibility and plausibility of the class in the evidence theory framework. The training set is complete, which means that the training data contain samples from all classes. Both measures lead to the same decision *ε*:

(30) $$\varepsilon \left(D_{s}\right)=\max m^{s}\left(\left\{\omega_{q}\right\}\right)$$

where $\varepsilon (D_{s})$ is the class label assigned to $D_{s}$.

By using the D-S evidence theory, the sensor nodes are classified into different classes. The relativity between the data stored in the sensor nodes is expressed by the symbol *δ*. Sensor nodes with more relevant content are classified into the same class. We provide the training set for the sensor nodes to be classified, and the set includes all sensor node classes. By exploring the data relativity, we use D-S evidence theory to combine the BPA to get a joint one and deduce the belief function. Assigning a sensor node to a specified class is determined by choosing the maximum belief value, which is beneficial to fully utilize the function of data fusion and other processes.

## 5. Content-Based Multi-Channel Network Coding Algorithm

In this section, the description of the CMNC algorithm is given in detail. The sensor nodes in the millimeter-wave sensor network are divided into different classes according to the analysis result of data content during the classification period while the sensor nodes in the same class are assigned the same channel for data transmission. In the transmission period, the sensor nodes execute both data fusion and network coding to decrease the network overload and improve throughput by eliminating the unnecessary data transmission. This section is divided into four subsections to describe the content and execution of the CMNC algorithm.

### 5.1. Assumptions

Without loss of generality, we first make assumptions to ensure that the proposed CMNC algorithm can run successfully.

- Each sensor node has a unique identification, which represents the logical address of the sensor node in the millimeter-wave sensor network, and it can be replaced by the MAC address.
- The millimeter-wave sensor network is fully connected, which means a sensor node can find a route to any other sensor node. There is no obstacle that the data transmitted in the network face. Each sensor node can be a sender and can receive data from all other sensor nodes when they use the same channel.
- Each sensor node has a label *L* to represent its class, and the change of the class depends on the data content. A sensor node only belongs to one class within a certain period.
- The millimeter-wave sensor network is a homogeneous network, such that the sensor node’s computing capacity, power consumption, communication cost, coding cost, transmission distance and propagation loss are all the same.
- Each sensor node has the ability to access multiple channels. However, the sensor node has one antenna, which means it can take up only one transmission channel in the corresponding time.
- The time that the sensor node occupies a channel is arranged according to the total length of the transmission data. However, in a unit of time, each sensor node can only send or receive a packet. The data that are not transmitted within the prescribed period need to wait until the next time.
- The sender knows the route in which the data were transmitted to the receiver, including through how many relays and which relay sensor node executes coding or forwarding of the data. A sensor node has a neighbor list to record the data packets obtained by their neighbor sensor nodes. A sensor node receives a packet that corresponds to the position value of one, otherwise it corresponds to zero.
- All sensor nodes in the network have the ability to process the data from senders or relays, which implies that each sensor node can encode or decode the data according to the transmission requirement.

### 5.2. Channel Assignment for the Sensor Nodes

There are multiple available channels for data transmission between the sensor nodes, and the sensor nodes can occupy one or more channels. However, the number of sensor nodes is far greater than that of the channels. How to allocate these channels for the sensor nodes becomes very important for utilizing the precious channel resources. The suitable channel assignment can maximize the efficiency of data transmission in the network. For the millimeter-wave sensor network, we assign different channels based on the classification results of the sensor nodes. This section explains the channel assignment for the sensor nodes.

We assume that the millimeter-wave sensor network includes channels $C=\{C_{1},C_{2},\ldots ,C_{N}\}$, where *N* is the number of channels, and the bandwidths of these channels are all equal. In the millimeter-wave sensor network, we only need to consider how to efficiently assign these channels to different classes. Other co-channel interferences and overlapping channels are not within the scope of our consideration.

The sensor nodes are divided into different classes according to their data content, and the number of classes also varies with the data content. We assume that there are *N* available channels and *M* classes in the network. If N⩾M, the number of the channels is more than that of the classes. We suppose channels in the network have the same bandwidth; the channels are arranged in a queue and to be assigned to the sensor nodes in turn. The last assigned channel is moved to the final position in the queue for the next assignment. There is no case that no available channel can be assigned when a sensor node requests transmission.

If N<M, the number of channels is less than that of the classes. There are no available channels in the network to which some sensor nodes can be assigned, which causes these sensor nodes to wait for the channel. The limited channel resources must be assigned carefully. A *high response priority assignment method* is designed to solve this problem. The length(x) is defined as the total length of the data that need to be transmitted for each class. Considering that the transmission time changes according to the different data lengths, the channel occupancy time *T* is proportional to the length(x). With the growth of the data length, the channel occupancy time of the sensor nodes in this class increases correspondingly. The channel occupancy time *T* is:

(31) $$T=length\left(x\right)/V+T_{C}$$

where *V* represents a sending rate of the channel in the millimeter-wave environment and $T_{C}$ represents transmission time in the channel. Due to the sender already knowing the data transmission route in the class, $T_{C}$ is known during channel assignment. When a sensor node requests a channel, the first step is defining the priority of the class to which it belongs. We introduce a dynamic priority assignment method; the priority of class increases with the waiting time. The class with the longer waiting time is more easily assigned an available channel. The change rule of the priority is:

(32) $$\mathrm{Priority}=\frac{T_{waiting\;time}+T}{T}$$

If these classes have the same waiting time, a shorter transmission time means higher priority. The class with a shorter transmission time has the priority to access the channel. On the other hand, the priority depends on the waiting time when these classes have the same transmission time. A longer waiting time means higher priority. Relative to the same transmission time of these classes, it utilizes the method of first come first serve. For longer transmission times of the class, its priority increases with the waiting time. When a class waits long enough, it gets very high priority, which ensures it an available channel. This method considers not only the channel assignment of short transmission time, but also the request sequence; it promises the class with a long transmission time that it can occupy the channel. Therefore, the method achieves a good compromise.

By using the channel assignment for different classes, any class of the sensor nodes can occupy an available channel for data transmission, and the time of occupying the channel is decided by the length of the data.

### 5.3. Network Coding Mechanism in the Process of Transmission

Network coding has been shown as a substantial increase in throughput in both multicast and unicast sessions via a wired and wireless network. The basic idea of network coding is that relay nodes combine several data into one packet or broadcast to the various receivers. Each receiver can decode the encoded data through obtaining some of the information from another node or extract an encoding vector from the encoded data. Thus, a relay sensor node can transmit more data by one packet, which ultimately increases the overall throughput and reduces the cost of forwarding.

**Figure 3:**
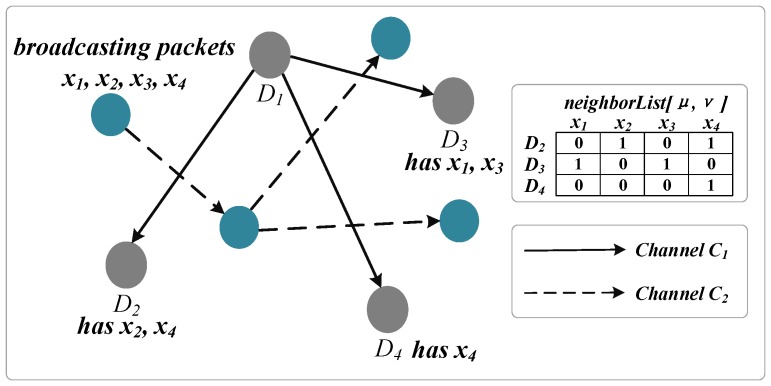
Example with a broadcast source and three receiver sensor nodes.

In this work, we use random linear network coding to further improve the data transmission in the millimeter-wave sensor network. It was first proposed in 2006 [34]. The core idea of RLNC is using the computing power of the sensor nodes. The sending sensor node combines different packets with linear encoding, and the original information packets are restored by calculation at the receiving sensor node after obtaining sufficient linear encoding packets. For example, the sensor nodes $D_{1}$, $D_{2}$, $D_{3}$, $D_{4}$ are assigned the same Channel 1 as shown in Figure 3, where sensor node $D_{1}$ needs to send the data packet $x_{1},\;x_{2},\;x_{3},\;x_{4}$ to $D_{2}$, $D_{3}$ and $D_{4}$. Each receiver has partial data, such as $D_{2}$ has the data $x_{2},\;x_{4}$, $D_{3}$ has the data $x_{1},\;x_{3}$, $D_{4}$ has the data $x_{4}$. Each sensor node has a neighborList[μ,ν] to record the data contained in the sensor nodes. neighborList[μ,ν] consists of two parts where *μ* is the sensor node number and *ν* is the data number. In Figure 3, the sending sensor node needs to broadcast four times by using the traditional methods. With the help of the RLNC, the sensor nodes have the ability of network coding, and the random coding vector from GF to the code combination $X_{1}=\varsigma_{1}x_{1}+\rho_{1}x_{2}+\tau_{1}x_{3}+\upsilon_{1}x_{4},\;X_{2}=\varsigma_{2}x_{1}+\rho_{2}x_{2}+\tau_{2}x_{3}+\upsilon_{2}x_{4},\;X_{3}=\varsigma_{3}x_{1}+\rho_{3}x_{2}+\tau_{3}x_{3}+\upsilon_{3}x_{4}$ can be used and broadcast, where GF is the *Galois field*. After the receiver gets three coding combination packages, it can adopt a linear operation to get the original packets $x_{1},\;x_{2},\;x_{3},\;x_{4}$. Compared to the traditional method, the sending sensor node only needs to broadcast three times to finish the transmission by using the RLNC.

The sensor node $D_{2}$ receives three code packages from $D_{1}$ and combines them with the source packet $x_{2}$ into the encoding vector matrix. Based on the Gaussian elimination of the matrix, the receiver can obtain data $x_{1}$, $x_{3}$ and $x_{4}$. The encoding vector matrix of $D_{2}$ is:

(33) $$\left\{\begin{array}{ccccc} 0 & 0 & 0 & 1 & x_{4}\\ \varsigma_{1} & \rho_{1} & \tau_{1} & \upsilon_{1} & X_{1}\\ \varsigma_{2} & \rho_{2} & \tau_{2} & \upsilon_{2} & X_{2}\\ \varsigma_{3} & \rho_{3} & \tau_{3} & \upsilon_{3} & X_{3} \end{array}\right\}⟹\left\{\begin{array}{ccccc} 1 & 0 & 0 & 0 & x_{1}\\ 0 & 1 & 0 & 0 & x_{1}\\ 0 & 0 & 1 & 0 & x_{2}\\ 0 & 0 & 0 & 1 & x_{4} \end{array}\right\}$$

Through the use of network coding, the sensor nodes combine the different data with a random coding vector before transmission, which further reduces the network overload and improves the network throughput without affecting the network function.

### 5.4. Algorithm Description and Analysis

The CMNC algorithm aims to improve the performance of data transmission for the millimeter-wave sensor network, which is different from the traditional methods. The biggest difference with other algorithms is that the sensor nodes are classified based on the result of exploring the data content. The sensor nodes belonging to the same class are assigned the same channel. During the data transmission, data fusion and network coding with the help of RLNC are both processed by the sensor nodes to improve transmission throughput. According to the data contents, the sensor nodes are divided into different classes and the sensor nodes belonging to the same class have the relevant data content. A fusion-driven model based on D-S evidence theory is designed to classify these sensor nodes, and each sensor node has its own class label *L*. The data content *δ* stored by the sensor node is compared to that of other sensor nodes, which has been classified in the training set (ϑ,L). Then, get the combined function of the BPA and the belief function. By using the belief function judgment, the maximum belief function $\varepsilon (D_{S})$ from Equations (28) and (30) is chosen as the basis of classification.

**Figure 4:**
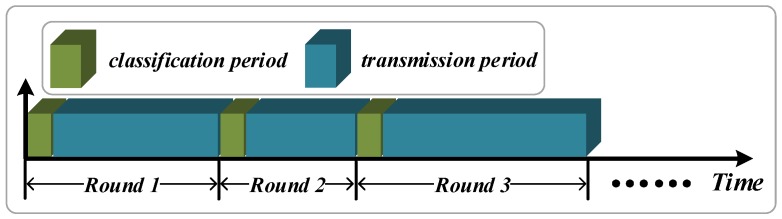
The round of the classification period and transmission period.

The operation of the CMNC algorithm is divided into continuous rounds. Each round consists of the classification period and the transmission period, as shown in Figure 4. The time of the classification period is planned by the number of sensor nodes requiring classification, and the data transmission time determines the length of the transmission period. In the classification period, all of the sensor nodes are classified according to the result of data content analysis, while the channels are assigned to the sensor nodes. The sensor nodes in the same class share the same channel. In the transmission period, the sensor nodes transmit the sensory data and complete the functions of both the data fusion and the network coding. As the destination of data collection in the millimeter-wave sensor network, the sink node receives all of the sensory data and is responsible for further processing the data. Considering that the classification performance is affected by the change of data content, the CMNC needs to re-classify the sensor nodes if the sink node detects a higher proportion of redundant data, which illustrates the low utilization of data fusion and network coding. Therefore, the duration of the transmission period is not the same in different rounds, and the proportion of redundant data can be set according to the actual application environment. Due to the number of classes in the network being uncertain, the relationship between the number of the channels and the classes is considered during the assignment. For the different situations, the CMNC provides different methods. When the number of channels is more than that of the classes, the CMNC assigns the channels sequentially, and the method of first come first serve is adopted. When the number of channels is less than the number of classes, the CMNC uses a high response priority assignment method from Equations (31) and (32), which can fully take into account the situation with numerous requests occurring in the channel. Waiting time and the total transmission time are the important factors of judging priority.

In the CMNC algorithm, the sensor nodes are classified based on their data content in order to utilize the function of data fusion in the transmission period. Data fusion can effectively decrease the network overload by eliminating redundant data in the millimeter-wave sensor network. The sensor nodes with more related data are classified into the same class and assigned the same channel in the CMNC algorithm. In this way, the function of data fusion can be maximized in the process of data transmission. In addition, the CMNC algorithm also adopts random linear network coding to further increase the throughput in the transmission period. The RLNC combines the original data *x* to get a linear combination. Each data block processes a combination with the random coding vector to generate a new linear combination package *X*, which needs to be forwarded by the relay node. For a sensor node, it decodes the received package *X* to get the original data. The main idea of network coding is to allow the sensor nodes to generate and transmit combination packages, then restore the original data from the combination packages. These simple operations performed on the sensor nodes result in a decrease in the delay of data transmission. Network coding also increases the robustness to packet losses. Therefore, the proposed CMNC algorithm is able to improve the performance of data transmission. The algorithm description is given in Algorithm 1.

**Algorithm 1 CMNC****Input:**    data content *δ*,    the channel set $C=\{C_{1},C_{2},\ldots ,C_{N}\}$,    the class set $\omega =\left\{\omega_{1},\omega_{2},\ldots ,\omega_{M}\right\}$**Output:**    the transmission strategy in the millimeter-meter network  1:  **for** the sensor node $D_{S}\in D$
**do**  2:   **for** the sensor node data *x*
**do**  3:       extract data content *δ* from the sensor node  4:   **end**
**for**  5:  **end**
**for**  6:  **for** the sensor node $D_{S}\in D$
**do**  7:   **for** the data content *δ*
**do**  8:       compare to the training set (ϑ,L) using Equations (18), (19), (20)  9:   **end**
**for**10:  **end**
**for**11:  calculate the combination of the BPA and the belief function of the sensor node $D_{S}$ by Equations (24), (25)12:  **for** the sensor node $D_{S}\in D$
**do**13:   **for** the data content *δ*
**do**14:       get the maximize belief function as the basis of assigning class by Equation (28)15:   **end**
**for**16:  **end**
**for**17:  get the class label $\varepsilon (D_{S})$ by Equation (30)18:  **for** the class set $\omega =\left\{\omega_{1},\omega_{2},\ldots ,\omega_{M}\right\}$
**do**19:   **for** the channel set $C=\{C_{1},C_{2},\ldots ,C_{N}\}$
**do**20:       compare the number of channels and classes21:       **if**
M>N
**then**22:             running the high response priority assignment method23:             calculate the Priority of the class by using Equation (32)24:       **else**25:             sequentially assigned to channel26:       **end**
**if**27:   **end**
**for**28:  **end**
**for**29:  assign the channel to the corresponding class30:  **for** each class *ω*
**do**31:   **for** the sensor node *D* belongs to the same class **do**32:       get the neighborList33:       **if** neighborList[μ,ν] = 1 **then**34:             some data have been gotten35:       **else**36:             some data have not been gotten37:       **end**
**if**38:   **end**
**for**39:  **end**
**for**40:  obtain the data to be transferred $x_{1},\ldots ,x_{n}$41:  generate a random linear encoding package $X_{i}=\varsigma x=\varsigma_{1}x_{1}+\varsigma_{2}x_{2}+,\ldots ,+\varsigma_{n}x_{n}$42:  **for** each class *ω*
**do**43:   **for** the sensor node *D* belong to the same class **do**44:       transmit the linear encoding package45:       **if** the receiver gets enough $X_{i}$
**then**46:             decoding package and gets the original data $x_{i}$47:       **else**48:             continue to receive $X_{i}$49:       **end**
**if**50:   **end**
**for**51:  **end**
**for**

Compared to the traditional methods, the proposed CMNC algorithm has a significant effect of both decreasing network overload by reducing redundant data and mitigating network congestion. In the traditional methods, the conventional method does not classify the sensor nodes according to the relationship of their data content, and the channels are assigned randomly for these sensor nodes. As shown in Figure 1a, $R_{1}$, $R_{2}$ are the relay nodes of the route to the sink node. The sensory data generated by $D_{1}$ has a high relevancy to that from $D_{3}$, and these data are sent to the sink node. The sensor nodes $D_{2},\;D_{4}$ have the same condition. In this way, the sensor nodes in the network occupy the channel randomly, and the relevant data cannot be processed by the relay nodes. The sink node receives many redundant data because of the low performance of data fusion and network coding with uncorrelated data at the relay nodes. Therefore, unnecessary network overload is caused by the traditional methods, which are not optimal choices as required. As a typical multi-hop network, data transmission in the millimeter-wave sensor network needs to wait for the channel assignment and routing establishment, which brings additional computing, communication and time overhead in the classification period. However, the CMNC algorithm can fully utilize the functions of data fusion and network coding to improve data transmission by reducing the network overload in the transmission period, which is much longer than the classification period. In Figure 1b, the sensor nodes are classified according to their data content and occupy the channel in order with the class. The relay nodes $R_{1}$ and $R_{2}$ encode and fuse the relevant data from $D_{1},\;D_{3}$ and $D_{2},\;D_{4}$, respectively. After network coding and data fusion, the relay node generates and sends the new data to the sink node, which decodes the data packet after receiving. The sensor nodes in other classes also complete data transmission in the same way, which effectively reduces both the transmission delay and the network overload compared to the traditional methods. Specifically, the channel assignment of the CMNC decreases the possibility of the transmission loss caused by the communication collision.

## 6. Simulations and Results

This section discusses the results of simulations to evaluate the performance of our proposed CMNC algorithm by comparing it to other algorithms. Both the sensor node classification model and the channel assignment model are analyzed at different scales of millimeter-wave sensor network scenarios.

### 6.1. Performance of the Fusion-Driven Model Based on D-S Evidence Theory

In the previous section, we used the D-S theory based on the data content to classify the sensor nodes. There are some simulations to verify the effectiveness of the fusion-driven model. The relevant parameters are determined, and $\phi_{q}$ is assumed to be the exponential function. The *θ* and *γ* are calculated according to the previous data. The results are obtained with θ=0.9, and *γ* gets a value of $1/\delta_{q}$, where the $\delta_{q}$ is the value of data content belonging to two training sets of the same class. *β* is found to have less effect on the result, which is assumed as β=2.

**Figure 5:**
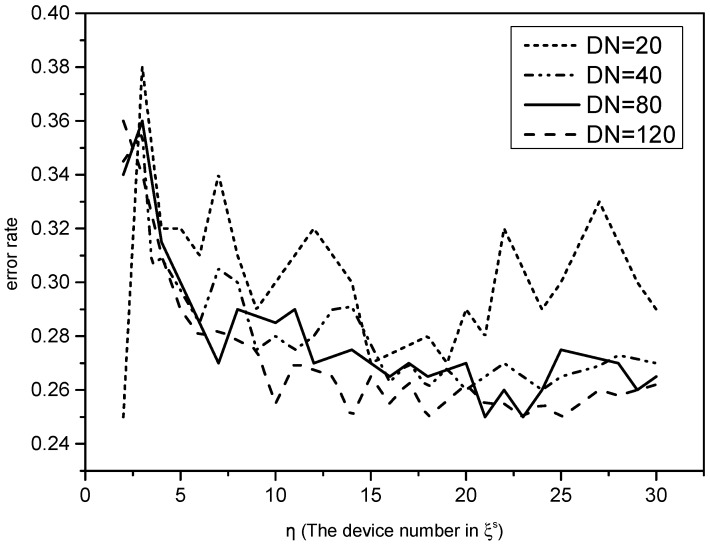
The error rate of the fusion-driven model based on Dempster–Shafer (D-S) evidence theory with different training set numbers.

In our simulations, the different sensor node numbers of the training set DN are adopted as 20, 40, 80 and 120, respectively. As an independent variable, the value of *η* changes from one to 30. We choose the average result of 100 samples to detect the error rate in the classification process. The results are shown in Figure 5.

As can be seen from the simulative results, selecting a sufficient number of *η* is important to reduce the error rate. For all of the different numbers of the training set, the D-S evidence theory’s error rate has a significant change with the growth of *η*. Although the data have large fluctuations, they show a downward trend. The error rates are similar to the different numbers of the training set, because there are enough sensor nodes in the training set to reduce the volatility. It can be concluded that enough *η* can reduce the error probability of the D-S evidence theory.

### 6.2. Performance of the CMNC Algorithm

In this section, the performance of the CMNC is investigated from the network throughput of channel assignment and the average cost. Network throughput is the most important part of the multi-channel distribution, and it is closely linked to the quality of the data transmission, which increases with the enhancing throughput. The average cost of the system is another issue to consider. With the growth of time, the cost of transferring a block of data has a great influence on the system, and reducing the average cost to the integrity of the system is particularly important.

In this subsection, three different algorithms are established and compared to the CMNC algorithm. CANCOR (Channel Assignment and Network Coded Opportunistic Routing) [35] is a nonlinear model that considers the feature of cognitive radio networks for the joint opportunistic routing and channel assignment problem. LACAV (Learning Automata-based Channel Assignment in Vehicular ad hoc networks) [36] is a channel assignment algorithm about learning automata and reusability for data transmission. RNC-AucCA (Random Network Coding Auction-based Channel Assignment) [37] is a joint algorithm with random network coding, channel assignment and opportunistic overhearing to maximize network throughput.

In the simulation, we assumed that 100, 200 and 400 sensor nodes are randomly distributed in the millimeter-wave sensor network. Every sensor node has the ability to choose the channel and to detect the neighbors. Due to the millimeter wave frequency being different from traditional wireless radio frequency, the simulation needs to consider the impact of the propagation loss. The main interferences of the transmission are the atmosphere and rain, summed up by the paper [4]. In our simulation, we do not consider the rainy environment, but only consider the influence of propagation loss caused by the atmosphere. In the short distance zone (200 m), the propagation loss of millimeter-wave in the atmosphere is similar to the normal radio transmission. After referring to many papers [4,5,6] about the millimeter-wave, the propagation loss in our simulation is 0.012 dB in the short distance zone (200 m). Before the start of the transmission, the D-S evidence theory is implemented to classify these sensor nodes into different classes.

**Figure 6:**
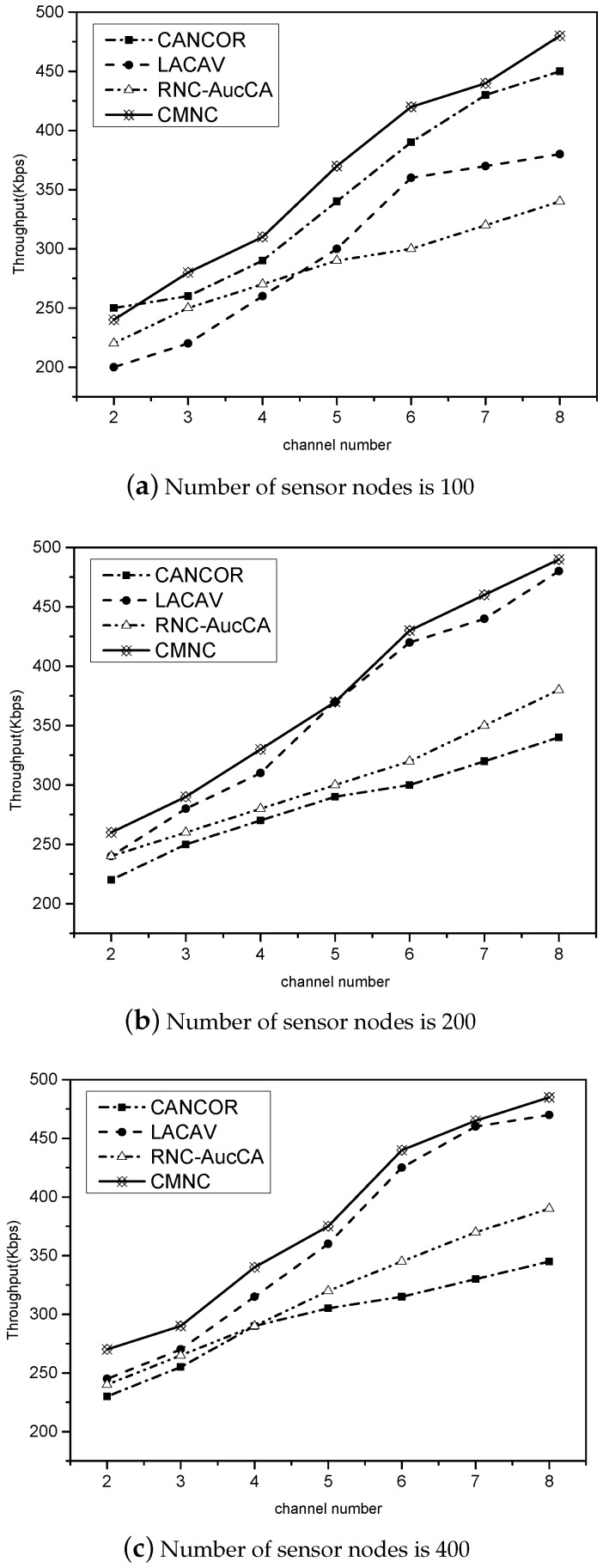
The relationship between the throughput and the number of sensor nodes.

The adjustment of the network throughput is affected by the number of available network channels. In the simulations, we change the channel number from two to eight. As seen from Figure 6, each algorithm’s channel throughput increases with the number of distribution channels and shows a trend of rising with different levels. When the number of channels in the CMNC algorithm is four times greater than before, the network throughput presents double growth. The growths of other algorithms are all weaker than that of the CMNC. The maximum throughput is also varied with the different numbers of sensor nodes. More sensor nodes in the millimeter-wave sensor network under the same number of channels lead to a greater network throughput. It can be concluded that the CMNC algorithm has obvious benefits on the throughput, and it is also affected by the increasing number of sensor nodes.

**Figure 7:**
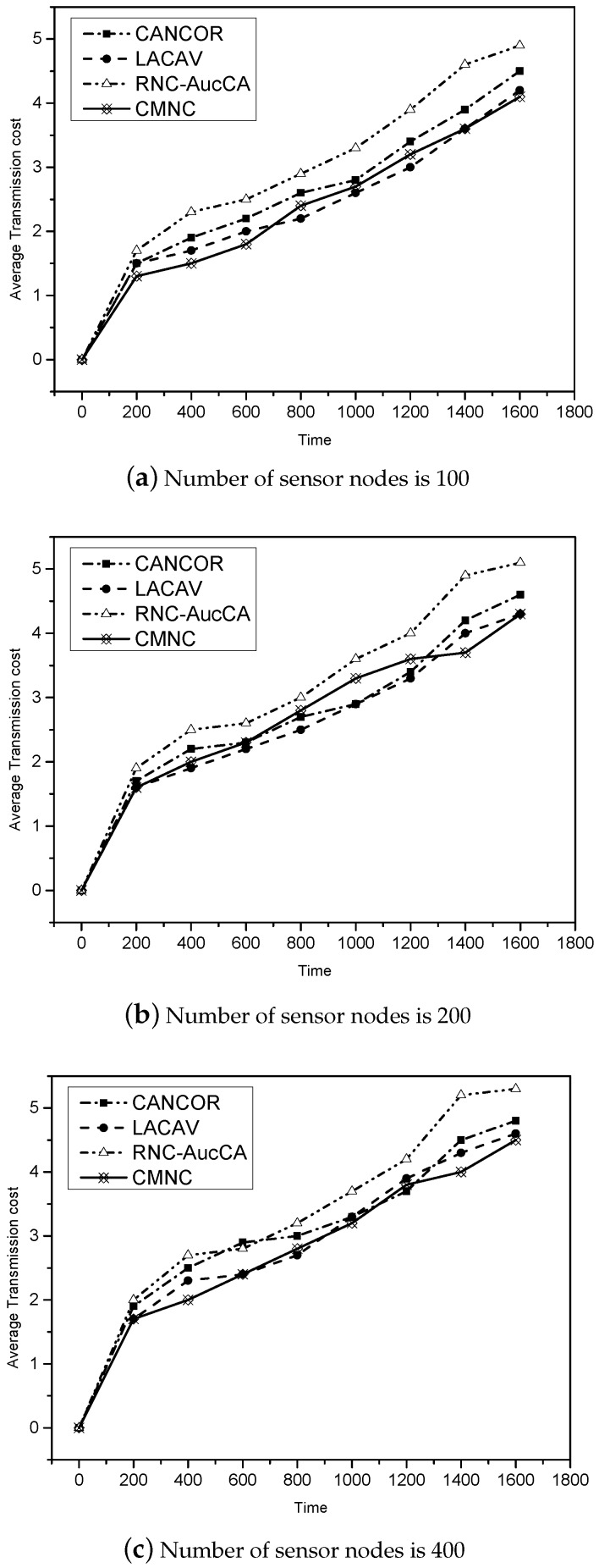
The relationship between the average transmission cost and the number of sensor nodes.

For the average transmission cost, we give the results of these four algorithms’ change over time. As shown in Figure 7, the average transmission cost grows with time. Comparing these four algorithms, the CMNC algorithm spends less average cost than the other three, which shows an obvious advantage. By using the different number of sensor nodes, transmission collisions increase with more existing sensor nodes. A lesser number of sensor nodes can obtain higher average transmission cost in the millimeter-wave sensor network.

The proposed CMNC algorithm utilizes data fusion and network coding to improve the network throughput of the transmission and to reduce the network overload. Compared to the traditional methods, the CMNC algorithm needs similar overhead to exchange global information in the classification period. Since the classification period is much shorter than the transmission period in the CMNC algorithm, such overhead is acceptable and has a small effect on scalability. For the introduced function of network coding in the CMNC algorithm, the overhead comes from the operation of encoding and decoding, which is determined by the GF. The size of GF is associated with the number of encoding packets. Both the data fusion and the network coding cause computational overhead, but effectively decrease the communication cost of the network. To further evaluate the scalability of the CMNC algorithm, different numbers of sensor nodes are adopted in our simulation. The simulation results under different parameters all show the good scalability of the CMNC algorithm and the better performance compared to the other methods.

## 7. Conclusions

To achieve the high quality of data transmission in the millimeter-wave sensor network, this paper introduces a novel CMNC algorithm, including the function of sensor node classification and channel assignment. A fusion-driven model is firstly proposed based on D-S evidence theory to classify the sensor node effectively, which is capable of classifying the sensor node according to the data content. By using the result of the fusion-driven model, the CMNC algorithm selects optimal channels and uses network coding for transmitting data between the sensor nodes. Extensive simulations are performed to evaluate the CMNC algorithm. The results show that the CMNC algorithm can achieve a satisfying quality of data transmission and has the potential to transform into a practical technique in the millimeter-wave sensor network.
